# Management of Developing Anterior Malocclusion due to SupernumeraryTooth with Preventive and Intercep-tive Approach: A 1½ Year Case Study

**DOI:** 10.5005/jp-journals-10005-1064

**Published:** 2010-08-17

**Authors:** Divya S Sharma, HV Kambalimath, Naveen Reddy Banda

**Affiliations:** 1 Professor, Department of Pedodontics and Preventive Dentistry, Modern Dental College and Research Center, Gandhinagar Indore, Madhya Pradesh, India; 2 Associate Professor, Department of Pedodontics and Preventive Dentistry, Modern Dental College and Research Center Gandhinagar, Indore, Madhya Pradesh, India; 3 Senior Lecturer, Department of Pedodontics and Preventive Dentistry, Modern Dental College and Research Center Gandhinagar, Indore, Madhya Pradesh, India

**Keywords:** Anterior malocclusion, interceptive orthodontics, supernumerary tooth.

## Abstract

Variety of clinical complications occurs due to the presence of supernumerary teeth, especially mesiodens. It may result in impaction of one or both central incisors which in turn may lead to a variety of malocclusions. Timely intervention not only prevents malocclusion but also the time taken for corrective orthodontics. A complete case report of developing mesiodens’ tooth germ resulting in malocclusion including treatment in 1½ year period is presented.

## INTRODUCTION

Supernumerary teeth (ST) are those, formed excess in number of teeth compared to the normal dental complement. ST can be found in both primary or permanent dentition, though 5 times less common in the latter.^1^ Several etiological theories have been put forth to explain their development. These are atavistic theory, i.e. phylogenetic reversion; dichotomy theory, i.e. dichotomy of tooth bud; dental lamina theory, i.e. due to local independent hyperactivity of the dental lamina. DNA mutations and maxillofacial developemental anomalies/syndromes (e.g. Cleft lip/palate, cleidocranial dysplasia, gardner syndrome) also may be found to be associated with ST. Combination of the genetic and environmental factors, i.e. multifactorial etiology has also been suggested as ST are more common in the relatives of effected children than in general population.^2-4^

ST can be classified on the basis of the time of appearance and position in the arch (mesiodens, paramolar, postmolar or impacted); on the basis of shape (supplemental/rudimentary). ST may therefore vary from a simple odontome, a conical or tuberculate tooth, to a supplemental tooth resembling a normal tooth. The most frequent locations of ST are the premaxilla and the mandibular premolar regions. The prevalence of ST has been found in the range of 1 to 3%.^5^

Mesiodens, a ST found in midline of maxilla between the central incisors, is the most commonly found ST with the prevalence rate of 0.15 to 1.9%. Pediatric dentists often find one or both maxillary central incisors impacted due to its presence.^1^

A case report of tooth germ of mesiodens causing im-paction of 11 and developing malocclusion on same side, including its management and follow-up is presented here.

## CASE REPORT

**Fig. 1:**
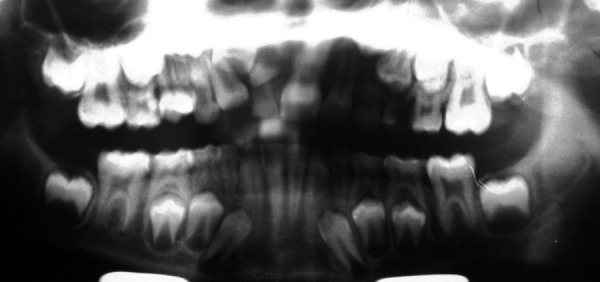
Preoperative orthopentograph showing severity of anterior malocclusion. Developing mesiodens can be seen apical to over-retained 51.11 can be seen impacted high in the alveolus, deviating developing root of 12 distally

A 8-year-boy reported to the Department of Pedodontics and Preventive Dentistry, Modern Dental College and Research Center, Indore, Madhya Pradesh (India) with the chief complaint of over retained 51 and noneruption of 11. History did not reveal any previous trauma in that region. Intraoral examination revealed vital over retained 51. 21 was erupted physiologically one year ago. Orthopentamograph revealed, a developing tooth bud of mesiodens occlusal to developing 11(Fig. 1). The mesiodens was inclined distally which had deflected developing 11 up in the alveolus in disto-occlusal direction which together with over-retained 51 forced the root of 12 distally and crown tipped mesially occupying the space ment for 11. Distally drifted roots of 12 had caused crowding in right maxillary area. Mandibular occlusion seemed to be alright at this time. 11 and 21 were in 8th stage according to Nolla’s stage of tooth development while the mesiodens in 5th.

## TREATMENT

Most of the times the mesiodens if not causing any pathologic changes in erupting permanent incisors, is left as such until apex of permanent teeth are closed.^6^ But if permanent tooth/ teeth has remained impacted for long time with the evidence of developing malocclusion, it is wise to intercept the condition.^7^

In this case after examining the radiograph, it was decided to extract 51 and to enucleate mesiodens expectating that after removal of hindrances impacted 11would erupt spontaneously (Fig. 1). As root of 11 was still immature, traction was not applied, for it might cause dilaceration and/or root resorption. Hence it was decided to allow the eruption of11 in distal direction only, which would be treated orthodontically afterwards according to the situation.

**Fig. 2:**
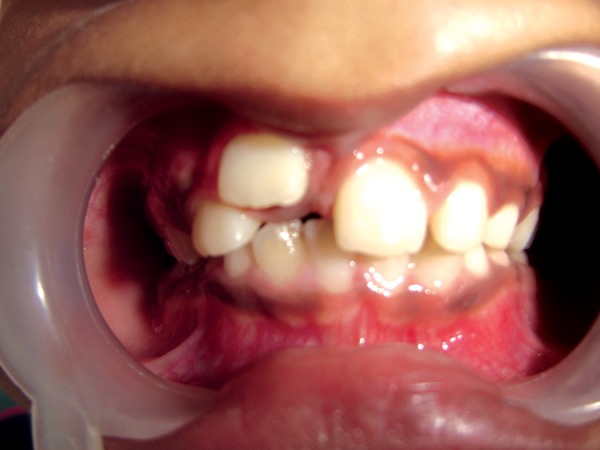
Front view showing, labially erupted 11 after 7 months

**Fig. 3:**
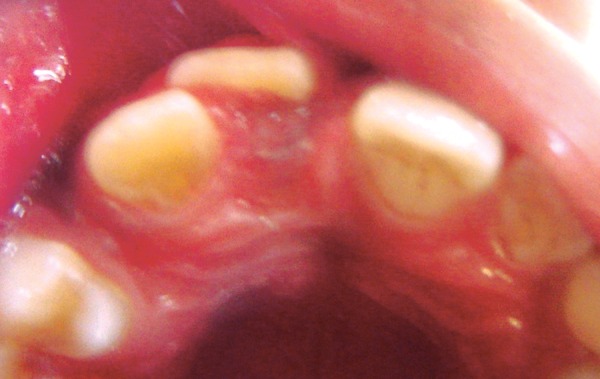
Occlusal view showing, amount of distal and labial displacement of 11

**Fig. 4:**
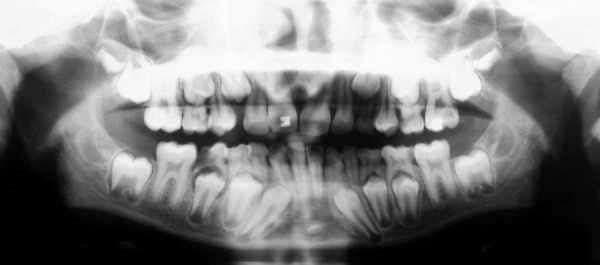
Orthopentogram showing, auto-correction in alignment of 12. 11, erupting well under guided orthodontic traction. Root formation of 11 & 12, almost completed

**Fig. 5:**
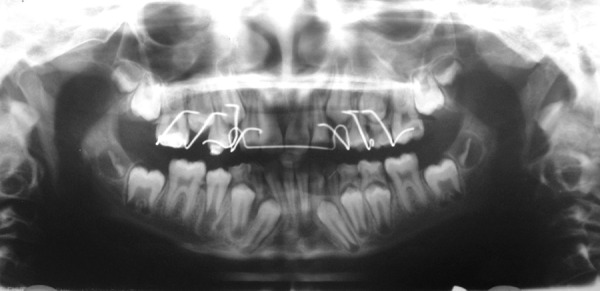
Post-treatment orthopentograph showing, well aligned maxillary anteriors. Both halves are exactly alike

**Fig. 6:**
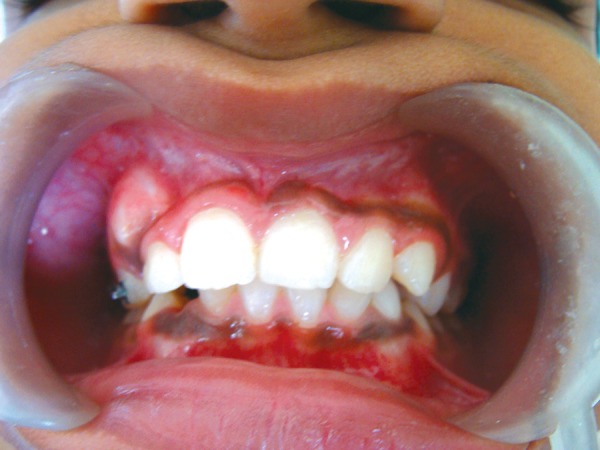
Post-treatment intraoral frontal view showing, resolved anterior malocclusion

Following hematological investigations, on scheduled date patient was given prophylactic antibiotic and analgesics one hour before the surgery. A full thickness flap was reflected and mesiodens was enucleated followed by extraction of 51. The flap was sutured back in place. Patient was advised to continue the regimen for five days. Patient was recalled after a week for suture removal followed by regular monthly visits. Approximately, after 7 months of surgery 11 could be seen emerging in oral cavity, distally inclined and labially displaced. A Hawley’s appliance with labial bow to restrain and guide labially erupting 11 in occlusal direction was designed in a way that it engaged incisal third of the erupting 11. No force was applied on 11 to mesialize it. It was expected that there would be autonomous mesial bodily movement of 11 while erupting as hindrances on mesial side were cleared and bone density still was less. After a period of 6 months 11 could be seen very well erupting occlusally with little mesial bodily movement of it (Figs 2 and 3). This time very light force was applied to mesialize it. 11 was bonded with bracket and a light force elastic was stretched from 11 to ‘U’ loop of Hawley’s retainer and labial bow activated. Patient was instructed to change the elastic every alternate day and to visit after one month. After about six months when repeated phone reminders made, patient appeared. This time 11 moved mesially drastically though not completely upto occlusion (Fig. 4). Case progression was overwhelming. The child and parents were very happy with the outcome. Perhaps because of improved esthetics parents lost their interest in visiting hospital. Same treatment was continued. Patient visited in May’ 08 with both central incisors in fair alignment. OPG showed complete root formation of 12,11 and 21with their roots similar to contralateral side in mesio-distal and vertical planes (Fig. 5). Clinically 12, 11 and 21 had no root bulge in alveolus proving good radicular labio-lingual position (Fig. 6).

## DISCUSSION

Many etiological factors may be there for delayed eruption of permanent maxillary central incisor. These include complete tooth agenesis (1.6-9.6% in permanent dentition); bone or mucosal barrier; presence of cyst related with impacted tooth/some other tooth/supernumerary tooth or predecessor tooth; dilaceration because of trauma to predecessor tooth; morphological and positional variants; over-retained primary incisor; disturbances in eruption related with several syndromes; lack of space in arch; most commonly, presence of supernumerary tooth; congenitally displaced tooth and ankylosis.^8^

Vitality of over-retained tooth and absence of bulge in the alveoli raised the suspicion of complete tooth agenesis of 11. The possibility of complete tooth agenesis for permanent central incisor is 0.00-0.01%.^9^ Though roentgenograph excluded this possibility. The presence of ST is the most common cause for delayed eruption of a permanent tooth, which was confirmed in roentgenograph. Delayed eruption of permanent teeth due to the ST is most common. Asians have slightly, greater than 3% of prevalence as compared to 1 to 3% in Caucasian population. Males are affected twice as more as females.^10^ Our case, an Asian male support these findings. The possibility of a syndrome, local and systemic conditions were ruled out with the clinical and medical history. It is conical or peg shaped ST which has been reported to be associated with the displacement of incisors. Di Biase had found that in case of delayed eruption, the affected incisor often has reduced root formation with an open apex; altered root curvature, labial displacement and inclination, as well as mesial or distal displacement/inclination.^11^ In the present case 11 had an open apex, labial displacement and distal inclination. The developing tooth germ of mesiodens was conical in shape. Our case supports his findings. The sagittal position of present case ST was within the arch (11.0%) and distally inclined (3.4%) as according to Gomes et al.^11^ Regarding vertical positioning 12.7% of the conical ST were located incisal to the crown as in this case. The incidence of overall ST in ‘crown under formation’ was 5.4%, whereas only 1.5% for conical ST proves the rarity of this case report.^12^

The ectopic positioning of immature 11 if not had been treated in time, would have led to root development molded to match the curvature of palate^13^ which may result in impac-tion of 11 making corrective treatment complicated.

Full thickness and perpendicular incision was the choice over semilunar incision. Many reports are there dictating maximum scar formation in the semilunar incision area. Scarred mucosa might had acted as a physical barrier to eruption of 11. Perpendicular incision heals without scar formation along with good visibility of surgical field.^14^

It is thought that the less time the normal eruption is delayed, the better the outcome. Approximately 54 to 74% of teeth have been reported to erupt spontaneously after removal of hinderance.^13^ This case report support the hypothesis that as long as there is sufficient space available, a tooth will erupt on its own.^10^ Spontaneous eruption was strongly expected in this case as 11 had immature apex and available space in the arch. During the root formation and restraining labial wire 11 was expected to move disto-occlusally with some of the mesial bodily movement.

At subsequent visit 11 was found to be completely erupted in oral cavity.

Root formation was almost complete and corono-radicular dentine thickness increased. Light but continuous orthodontic traction therefore was applied which resulted in successful outcome without any evidence of dilacerations, root resorption, tooth sensitivity or nonvitality.

## CONCLUSION

This case report presented the successful management of developing severe malocclusion in anterior region due to tooth bud of mesiodens. Early diagnosis, precise surgery and controlled approach were the key points. Case reports regarding commencement of treatment at such an early stage of tooth and mesiodens development was not found in literature. Surgical, preventive as well as interceptive orthodontics were all done in the department of pediatric dentistry. The case report also emphasizes the importance of esthetics after achieving of which the patient got reluctant to come.
